# MULTIDIMENSIONAL SOCIAL RELATIONSHIP AND LONELINESS AMONG OLDER ADULTS WITH COGNITIVE IMPAIRMENT

**DOI:** 10.1093/geroni/igae098.3318

**Published:** 2024-12-31

**Authors:** Soobin Park, Sojung Park, BoRin Kim, Takashi Amano, Jihye Baek

**Affiliations:** Washington University in St. Louis, St. Louis, Missouri, United States; Washington University in St. Louis, St. Louis, Missouri, United States; University of New Hampshire, Durham, New Hampshire, United States; Rutgers, The State University of New Jersey, Newark, New Jersey, United States; Washington University in St. Louis, St. Louis, Missouri, United States

## Abstract

As a modifiable risk factor of cognitive impairment, loneliness has received growing attention in research. By focusing on older adults with cognitive impairment, this study aims to add to the knowledge about the complex association among cognitive impairment, social relationships, and loneliness We explored distinct patterns of multidimensional social relationships and examined how these patterns influence social and emotional loneliness among older adults with cognitive impairments. Data came from the 2018 Health and Retirement Study and aged over 65 years. To focus on older adults with cognitive impairments, we excluded respondents with high cognitive function measured by the Telephone Interview for Cognitive Status (TICS) score (N=640). Latent class analysis was used to identify subgroups of social relations using multidimensional indicators of social relations including quantity (e.g., frequency of social contact with varying modes, social network size) and quality (e.g., positive and negative social support). Five patterns of social relationships emerged: Lack Friends/Weak (16%), Lack Children/Positive Friends (10%), Lack Family (11%), Virtual Diverse (52%), and Diverse/Negative (11%). Regression analyses showed Diverse/Negative group (p< 0.01) was significantly associated with a higher level of emotional loneliness compared to the Virtual Diverse group. All three groups (p< 0.05) except Lack Children/Positive Friends (p>.1) had significantly a higher level of social loneliness. Interestingly, more than half had a quantitatively diverse social relationship, maintaining rich social networks despite their cognitive impairment. Our findings highlight the importance of efforts that help decrease negative and secure positive or meaningful support as ways to reduce loneliness among cognitively impaired older adults.

